# Splenic Laceration: A Rare Complication of Abdominal Paracentesis

**DOI:** 10.7759/cureus.10749

**Published:** 2020-10-01

**Authors:** Ari Friedman, Adel Chergui, Ephraim Leiderman

**Affiliations:** 1 Medicine, Jacobi Medical Center/Albert Einstein College of Medicine, Bronx, USA

**Keywords:** paracentesis, splenic laceration, procedure, complication of treatment

## Abstract

Abdominal paracentesis is a commonly performed diagnostic and therapeutic procedure with a low complication rate. Previously described complications include injury to the abdominal wall, small bowel perforation, and abdominal hemorrhage. Splenic injury has also been described as a complication from bedside procedures including colonoscopy, upper gastrointestinal endoscopy, thoracentesis, and pleural biopsy. This case highlights a previously unreported complication from an abdominal paracentesis, splenic laceration.

## Introduction

Diagnostic paracentesis is a commonly performed diagnostic procedure. While complications are rare [1], they have been noted to occur and include injury to intra-abdominal organs and vascular complications [2]. Knowledge of potential complications of a procedure is essential both in providing informed consent as well as allowing the proceduralist to be vigilant in taking the precautions to avoid such complications.

## Case presentation

A 59-year-old female with a past medical history significant for hypertension, diet-controlled diabetes, HIV, severe tricuspid regurgitation, untreated hepatitis C virus, ascites, and end-stage renal disease receiving hemodialysis presented to the emergency department (ED) due to shortness of breath in the setting of missed dialysis sessions. For several weeks prior to hospitalization, the patient had worsening shortness of breath and increasing abdominal swelling, as well as some upper respiratory symptoms. On arrival, the patient was afebrile (temperature of 37°C), blood pressure (BP) was elevated to 182/84, but the patient was not tachycardic or dyspneic. Given the recent infectious respiratory symptoms and increasing ascites, a diagnostic paracentesis was performed in the ED. Documentation did not specify whether ultrasound guidance was used for the procedure, but revealed a left upper quadrant approach and the removal of 10 cc of bloody fluid. The sample was lost, and thus no further analysis was performed. After the paracentesis, the patient began complaining of left-sided abdominal pain, for which she was given topical anesthetic and then admitted to the medicine service for further management.

On admission to the medicine service, the patient continued to express concern for significant pain in her upper left abdomen that began after the procedure. At the time of admission, the patient remained afebrile, and had a BP of 164/92 and heart rate of 78 beats per minute. The patient’s abdominal exam was significant for exquisite tenderness to light palpation in all quadrants, precluding an assessment of splenomegaly and prompting the concern for a differential diagnosis including spontaneous bacterial peritonitis (SBP) or a complication from the prior paracentesis. An abdominal X-ray was ordered, which showed no free air under the diaphragm, and a repeat diagnostic paracentesis was planned to evaluate for SBP.

Using bedside ultrasound, a large pocket of peritoneal fluid on the right side was noted and marked for diagnostic paracentesis. Using sterile technique and ultrasound guidance, the paracentesis needle was observed to enter the fluid collection in the abdomen, without penetrating any adjacent structures. The fluid returned during the paracentesis had the appearance of frank blood, causing high concern for hemoperitoneum as a consequence of the initial paracentesis.

**Figure 1:**
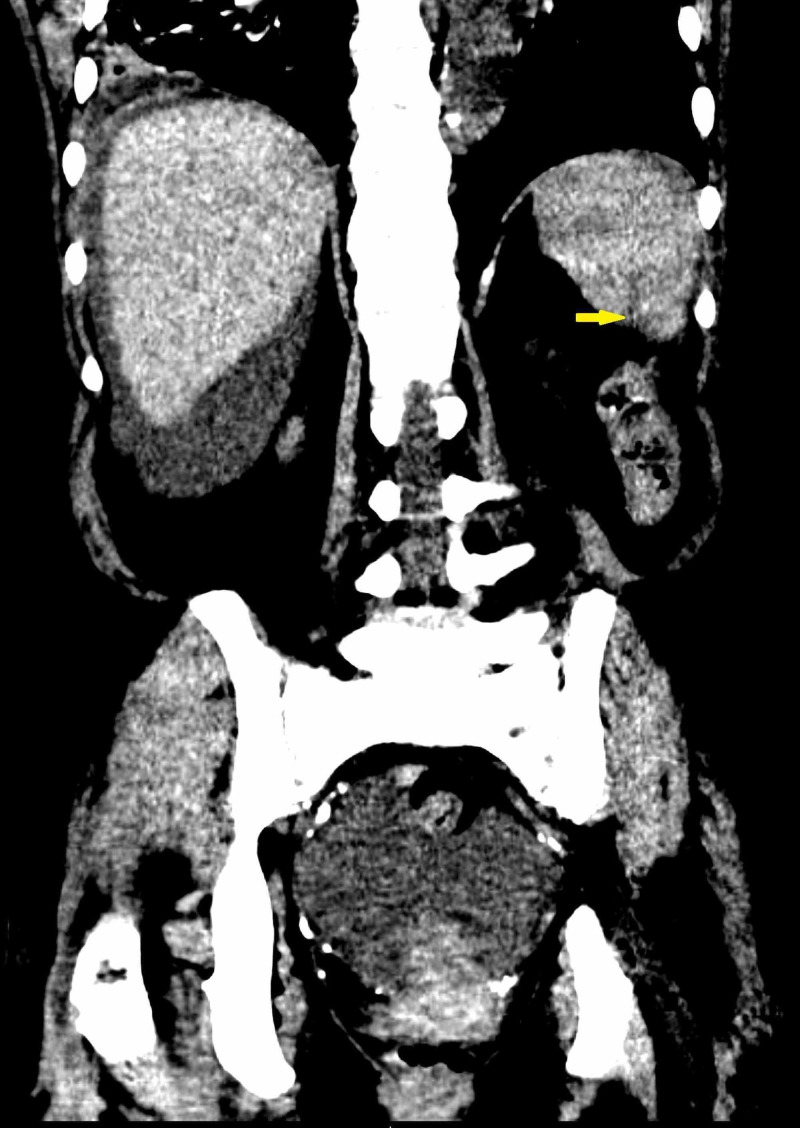
Splenic Laceration

An urgent CT angiography of the abdomen was ordered, which showed lacerations in the inferior splenic pole, consistent with a grade 1 splenic laceration, with no evidence of extravasation (Figure 1). An urgent surgical consult was placed, and the patient was transferred to the surgical ICU for further management. The patient had an uneventful stay in the ICU, remained hemodynamically stable, and did not require surgical intervention. There were no further drops in the hemoglobin level, and the patient did not require transfusion. The patient received hemodialysis for removal of excess fluid and remained afebrile and with improvement of the abdominal pain. Over the clinical course, there was continued improvement in abdominal pain, and the patient was eventually downgraded to surgical floors and then discharged from the hospital without any lasting complications or morbidity.

## Discussion

While injury to intra-abdominal organs is a known complication of paracentesis, splenic laceration has not been described as an observed complication in the literature. Splenic laceration is a rare but observed complication of a wide range of invasive abdominal and thoracic procedures [3]. The procedure most commonly associated with such injury is a colonoscopy, with upper gastrointestinal endoscopy also known to cause splenic injury [3]. Renal and pleural biopsy, nephrectomy, and thoracentesis have also been associated with splenic complications [3]. While paracentesis is a safe procedure with a low complication rate [1-2], previous case reports have described vascular complications including abdominal wall hematomas [1], small bowel perforation [4], and abdominal hemorrhage [5]. In general, when possible, the upper abdominal quadrants are avoided when performing paracentesis to avoid accidental laceration of the liver or spleen. Although this patient did not have any splenomegaly on imaging, the initial approach for the first paracentesis as done in the left upper quadrant placed the patient at risk for the resulting splenic laceration. The resulting grade 1 splenic laceration, while likely responsible for the observed hemoperitoneum and abdominal pain, was not a thermodynamically significant laceration, as reflected by its relatively small size and lack of observable impact on the patient's subsequent hemodynamics and hematocrit.

## Conclusions

This case represents a previously undescribed complication of paracentesis that providers should be aware of. Using ultrasound guidance for insertion of the needle as well as paracentesis kits with safety protected needles can reduce the risk of vascular complications or puncture injuries to intra-abdominal organs.
